# Supplementary material for: Are carbon emissions trading and green financial instruments synergistic? -Comprehensive quantitative research based on content analysis
Source: PLoS One. 2024 Mar 7;19(3):e0298601. doi: 10.1371/journal.pone.0298601 (PMC10919601; doi:10.1371/journal.pone.0298601)
Supplement: S1 Data — (ZIP) [file pone.0298601.s001.zip › Data/地级市Z_F工作报告.zip!/║┌┴·╜¡/║┌║╙╩╨/└┤╫╘╣1⁄2╓┌║┼í╛┬φ┐╦╩2╛▌═°í┐_2004.docx]

各位代表：

现在，我代表市人民政府，向大会报告工作，请予审议，并请各位政协委员和列席会议的同志提出意见。

一、2003年工作回顾

过去一年，在省委、省政府和市委正确领导下，全市人民团结拼搏，开拓进取，克服非典疫情和严重自然灾害的影响，实现了国民经济和社会事业全面发展。国内生产总值实现96.8亿元，同比增长7.2%。全社会固定资产投资完成24.3亿元，增长10.8%。社会消费品零售总额实现27.5亿元，增长8.1%。财政一般预算收入完成3.69亿元，增长1.4%。

经济结构调整取得成效。农业实现增加值15.5亿元。种植业播种面积830万亩，其中绿色食品面积157万亩，增长57.2%；大豆、马铃薯、经济作物、饲草、柞桑蚕“五大基地”面积349万亩，增长56.5%。畜牧业产值占农业总产值的19.7%，同比提高3.7个百分点。林业实现增加值6716万元，增长3.1%，完成造林18万亩。工业规模以上企业实现增加值5.4亿元，增长8.2%。国有工业企业改制面达92.5%，北安热力公司管网改造、151煤矿“一通三防”改造等17个技改项目投产，黑宝山煤矿、黑河热电厂等4个工业信息化项目完成。第三产业实现增加值46.1亿元，增长11%。边境旅游市场全面放开，共接待国内外旅游者47万人次，其中边境游8.1万人次，增长0.6%。五大连池国家级自然保护区被纳入世界人与自然生物圈保护区网络，国家地质公园日前被联合国教科文组织批准为世界地质公园。瑷珲历史文化旅游开发区完成总体规划，卧牛湖滑雪场确定投资主体。全市民营经济有较快发展，私营企业和个体工商户户数、从业人员、注册资金同比分别增长5.7%、6.6%、18.7%。新建和改造民营企业项目20个，38个项目纳入省民营经济发展项目库。全市一二三次产业比例由32.8：20.9：46.3调整为30.8：21.6：47.6。

重点项目建设顺利推进。积极争取国债资金和金融部门的支持，202国道、机场路建成通车，绥北公路完成路基和构造物工程，6条通县乡公路开工建设，其中讷五公路投入使用。黑河国际公路客运站正式开工，黑龙江大桥前期工作取得重要进展。富地营子水库实现蓄水，二门山水库完成大坝基础及上下游围堰浇筑。新兴基础原材料加工区完成“三通一平”，4户高耗能企业入区建设。铃田马铃薯颗粒全粉、嘉宇畜产品加工项目建成投产，北安完达山乳业、爱辉龙丹乳业、嫩江牛奶蛋白真丝等项目进展顺利。三精黑河药业GMP改造项目完成并通过国家认证。黑河热电厂三期工程投入运行。西沟抽水蓄能、乌二、三湾电站和翠宏山铁矿、关鸟河水泥厂等一批项目前期工作取得明显进展。全市列入省财源项目40个，开工建设27项，年内投产达效17项。

对外开放有新的进展。对外贸易进出口总额实现10902万美元。年初与俄阿州政府确定赴俄森林采伐、矿产开发、房屋建筑等22个合作项目顺利推进。引进外贸企业18户。互贸区扩大至市区得到省政府批复。开通黑河至雅库茨克临时货运包机航线。在全国率先进行银行间本币结算试点业务。重新规划口岸功能，改造旅检通道查验设施，旅贸通道分开、客货分检，实行12小时工作制，提高了验放速度。非典期间货运正常，客运在全省率先恢复通关。招商引资取得较大成果。共签约139项，合同总金额9.5亿元。落实项目137项，其中超千万元以上14项。实际到位资金50886万元，增长18%。

城乡建设步伐进一步加快。完成了城市近期建设规划、重点小城镇总体规划和重要区域详控规划编制。全市新开工建筑面积69.6万平方米，增长10.1%，其中市区21.7万平方米，增长11.8%。全市土地资产收益6198万元，增长5.9倍，其中市区3800万元，增长6倍。市区投入1516万元，对城市道路、排水等基础设施进行改造，火车站站前广场和王肃公园改造工程完工。启动“无烟城市”创建工作，小区并入集中供热和联片供热面积31万平方米。

各项社会事业全面发展。防治非典工作取得阶段性重大胜利。市第一人民医院住院部大楼主体工程完工，第二人民医院大楼投入使用，全市6所疾控中心启动建设。人口自然增长率控制在3.85‰，生育符合政策率达到98.49%。组织实施各类科技项目34项，其中国家和省级23项。适龄儿童入学率达99%，初中生巩固率达99.2%，中小学生辍学率控制在3%以下，高考录取率达77.1%。创建黑河学院工作扎实推进，45所中小学危房改造工程完工，教育大厦投入使用，教育信息平台开始运行。黑河市人民艺术剧院、群众艺术馆改扩建项目启动。竞技体育项目获奖牌90枚，3次打破省纪录。新上全民健身路径工程16处。民族、宗教、外事、侨务、老龄、档案、广电、文博图、地方志等工作都取得进展。

人民生活水平有一定提高。实现就业1.27万人，再就业2.64万人，分别超额完成省下达任务；下岗职工再就业率为44%，城市居民登记失业率为3.4%，均好于全省平均水平。职工工资和三条保障线资金发放率为100%。城乡居民储蓄存款余额104.6亿元，比年初增长7.5%。全市城镇人均住房建筑面积20.9平方米，增长6%。农村人均住房面积18.26平方米，住房砖瓦化率为60.7%。

社会秩序保持安全稳定。强化社会治安综合整治，破获北安“1•31”、合作区“2•09”等一批重特大案件，全市刑事案件发案总量保持全省最低水平。强化信访工作，妥善处置北安庆华厂等群众上访事件，重点案件时限内办结率100%。加大检查指导和整治力度，安全生产下滑局面得到有效遏止。努力抗击各类自然灾害，及时安置受灾群众，维护了正常社会秩序。森林防火工作取得较好成绩。边境秩序持续稳定。

政府自身建设不断加强。自觉接受市委领导，认真贯彻落实市委重大决策。主动接受人大政协监督，积极配合人大代表和政协委员对5个政府部门进行评议，办理代表建议29件，委员提案147件。进一步转变政府职能，行政审批事项由年初225项减到147项，取消或降低行政事业性收费26项。积极开展政府系统“两风”建设，查摆出的54个问题基本解决。推进依法行政、廉洁从政、高效施政、监督检查4项长效机制，开展专项检查12次。狠抓工作落实，共督办事项970项。市政府班子成员深入基层，调查研究，撰写调研文章18篇，现场办公261次。推行“六项制度”，扩大了“一条龙”办公、“一站式”服务范围。

各位代表，2003年政府各项工作取得一定成绩，得益于省委、省政府和市委正确领导，得益于市人大、市政协和各民主党派认真监督和积极支持，得益于中省直部门大力配合，得益于驻黑河解放军和武警部队无私奉献，得益于全市人民辛勤努力工作。在此，我代表市政府表示衷心感谢，并致以崇高敬意！

在肯定成绩的同时，我们也要清醒看到，全市经济社会生活中仍存在一些矛盾和问题，加快发展还面临许多困难。主要是：国内生产总值、财政收入和城乡人均收入低，与发达地区差距逐渐拉大。由于遭受历史罕见自然灾害，粮食总产量降至20年来最低水平，农业增加值下降10.4%，农民人均纯收入1830.9元，降至10年来最低点。我市尚处于工业化初期，工业总量小，科技含量低，缺少拳头产品，机制不活。经济外向度低于全国和全省平均水平，受非典和黑龙江枯水等多种因素影响，外贸进出口总额下降23.3％。财政减收因素多，刚性支出增加，职工工资和三条保障线支出压力很大。工作上，在解放思想、转变作风和抓落实等方面还存在一些突出问题，经济发展环境还有待进一步改善，等等。对此，我们必须高度重视，认真研究，努力解决。

二、2004年工作安排

2004年政府工作总体要求是：认真贯彻十六届三中全会、中央和省委经济工作会议及市委二届十三次全会精神，牢固树立科学的发展观，高举“努力快发展、全面建小康”旗帜，积极实施旅贸牵动、工业立市、农业强市、科教兴市战略，抓住项目建设、招商引资和改善环境三个关键环节，促进经济快速发展和社会全面进步，为全面建设小康社会奠定坚实基础。预期目标是：国内生产总值增长8%，力争实现9%，其中一二三产分别增长6%、9%、9.2%以上。固定资产投资增长7%。社会消费品零售总额增长8%。人口自然增长率控制在5.6‰。围绕上述目标，重点做好十个方面工作：

（一）围绕增加农民收入，推进农村经济结构调整。把农业、农村和农民问题摆在突出地位着力解决好。农业增加值增长8%，农民人均纯收入增长15%。培育优势主导产业，突出发展六大经济。抓住国家政策向粮食主产区倾斜的机遇，继续抓好“五大基地”建设。优化种植业结构，改善作物品质，提升商品粮基地地位。深入实施大豆振兴计划，发展大豆经济。全面普及模式化栽培，重点推广行间覆膜技术，覆膜面积达到50万亩以上。按照“主辅换位”要求，发展牧业经济。加快推进肉牛养殖区、奶牛养殖区、绒山羊养殖带和绒山羊良种繁育场建设，牧业产值占农业总产值比重达25%以上。发挥生态优势，发展绿色经济。打造绿色食品统一品牌，形成规模优势，绿色食品监测面积达到200万亩。多元投入，多种经营，发展林产经济。继续实施森林生态效益补偿试点工程和退耕还林工程，建设林业生态和产业两大体系。大力开发种苗、经济林、食用菌、山野菜和北药产业，全市苗木、沙棘、桑树面积分别达到1万亩、10万亩和2万亩。以俄罗斯市场为导向，发展外向型经济。扩大逊克、北安、爱辉蔬菜出口基地规模，总面积达到5万亩。逊克、爱辉等境外农业开发区面积达到5万亩。根据实际需要转移农民，发展劳务经济。按照“公平对待、合理引导、完善管理、搞好服务”原则，抓好劳动力培训和用工对接，维护农民工合法权益，转移农村劳动力8万人，增长25%。壮大龙头企业，提升产业化水平。发挥区域优势和产业特点，积极发展乳品、肉制品、油脂、马铃薯、山野菜、亚麻、甜菜等农产品加工，北安完达山和恒泰、爱辉铃田和嘉宇、五大连池山口渔业等龙头企业实现达产达效，嫩江牛奶蛋白纤维争取建成投产。逊克和孙吴也要围绕羊绒、沙棘加工，新上几个带动能力强的企业。积极发展专业协会、股份合作社、农业公司等新型农民合作经济组织，培育营销主体，引导农民拓展市场，发展“订单农业”。提高农业科技含量，推进农业标准化、机械化生产。继续实施种养业良种工程，加快优良品种引进、繁育和推广，扩大良种基地规模。强化县、乡农技推广体系建设和农业科技示范园区建设。把农业机械化作为发展农业的治本措施，搞活更新机制，加快更新步伐。积极争取和推进农机作业公司试点工作，与中省直企业、单位齐心协力，共同推进农业机械化，以机械化促进标准化。强化基础设施建设，增强农业综合生产能力。努力争取农业综合开发基金和土地复垦基金，加大农田水利建设和农业综合开发力度，重点搞好抗旱水源、节水设施和防洪排涝工程建设，加速治理水土流失，加强耕地保护。健全农业信息网络，开展农业信息服务。继续深化农村改革，减轻农民负担。抓好农村税费改革，坚决落实中央和省一系列规定，确保农民负担控制在国家规定政策范围之内。在保护农民权益的基础上，加快农村土地经营权流转，促进土地规模经营。进一步深化粮食流通体制改革，搞活粮食市场。完善农村扶贫机制，重点抓好山区、老区扶贫工作。加强场县合作，促进区域经济一体化发展。各县（市）区要进一步加强同农垦北安、九三分局、中储粮公司以及部队农场、劳改农场合作，开展场县共建，发展县域经济。加强相互沟通，优化资源配置，促进城乡统筹发展。

（二）扩大产业规模，增强工业经济实力。坚持开发、改造和改革并重，依托资源优势，培育支柱产业，壮大工业经济。全市规模以上工业企业增加值增长8.5%。加速矿产和能源资源开发。抓住东北老工业基地振兴的有利时机，争取上一批大的项目。在开工建设西部年产60万吨煤矿基础上，对东部西岗子煤田和西部黑宝山—木耳汽煤田做进一步地质勘探和详查。实施煤转电工程，以黑河热电厂为核心，开工建设嫩江热电厂。培育水电支柱产业，继续推进西沟抽水蓄能、乌二、三湾、新鄂等水电站项目前期工作。争取嫩江多宝山地区、逊克宝山地区和五大连池矿泉水开发项目纳入国家老工业基地调整改造规划，积极推进翠宏山铁矿、东安岩金矿和富强岩金矿项目。加快企业技术改造。认真抓好24个技术改造项目。对三精黑河药业、金龙堂制药有限公司、北安飞龙动物制药有限公司等企业进行技术改造，扩大生产规模。积极引进先进技术和工艺设备，加快粮油加工业技术改造。积极引进战略投资者，把五大连池矿泉水产业做大做强。围绕农产品加工和矿产品加工，开发高技术含量、高附加值、高市场容量的新产品，不断延伸产业链条。积极推进企业改革。以建立现代产权制度为方向，全力抓好黑河市热电厂、黑河铁路（集团）公司等重点国有企业改革，实现多元持股。优化西沟水电有限公司、黑河双兴木业有限公司股权结构。上半年力争完成国有小企业民营化改造。抓好首钢庆华厂的破产重组工作，推进西岗子煤矿、多宝山铜矿实施政策性破产。

（三）以对俄经贸合作为重点，进一步扩大对外开放。坚持走出去与引进来相结合，建设新一轮对外开放的大通道、大窗口、大平台，全力构筑对外开放新格局。对外贸易进出口总额增长15%。推进园区建设，扩大经济技术合作。依托黑河经济合作区，加大入区项目策划、经营主体引入和基础设施建设力度，抓好新兴基础原材料加工区、出口加工区、木材加工区建设。协调俄阿州建立工业园区，吸引国内企业到园区从事加工、组装、生产，开展境外加工贸易。推进在俄阿州和赤塔州森林采伐和木材加工、阿州加里矿铁矿开采、对俄建筑承包等合作项目。调整贸易结构，努力扩大贸易额度。边境小额贸易要以市场为导向，稳定旧钢轨、木材、果蔬等长线大宗产品进出口，增加铝锭、柴油、有色金属、化工产品、机电产品等高附加值产品进出口。提高出口产品质量，向俄腹地延伸，提高市场占有率。积极推进民贸公司化运作。促进大黑河岛功能转化，抓好《黑河中俄边民互市贸易区管理暂行办法》实施工作，加强边民互市贸易的组织、管理、协调和服务。利用中外各种宣传媒介，全面宣传互贸区政策及相关情况，扩大知名度，吸引人流物流。进一步扩大一般贸易规模，北安、嫩江、孙吴要增加向韩国、日本等国家出口额度，其他地方也要尽快实现突破，增强县域经济外向度。引进和扶强外经贸经营主体，促进对外贸易规模不断扩大。加速通道建设，提高口岸服务水平。把建设黑龙江大桥作为重中之重，全力推进。继续抓好孙逊、沾五等通县乡公路建设，力争年内完工。积极推进111国道和嫩江至黑宝山公路改造工程。争取国家批准黑河航空口岸，并开通至俄罗斯国际航线。组建国际汽运集团，发展集装箱运输业。推进黑河国际物流中心建设，构建物流基地。加强口岸基础设施建设，合理设置口岸功能区，加速货运汽车轮渡码头西移。引入竞争机制，打破报关、航运、汽运等方面垄断局面。加强与俄方协调联系，完善定期会晤制度和重大问题沟通协商机制。严格执行口岸降费政策，坚决取缔口岸乱收费和不合理收费项目，协调俄方同步降费。

（四）抓好景区建设和市场开拓，促进旅游业健康发展。全面实施旅游发展总体规划，加快国内游与边境游有效对接。接待国内外旅游者增长49%，其中边境游增长48%。推进旅游项目建设。按照“五区三线”发展框架，尽快完成各县（市）区旅游发展规划的编制。推进18个旅游项目，重点抓好瑷珲历史文化旅游区、五大连池保健康疗旅游区、卧牛湖休闲度假与冰雪运动健身区、沾河漂流旅游区、山口旅游度假区等景区建设，确保黑河远东国际滑雪中心年底正式运营。积极争取五大连池风景名胜区4A级、瑷珲历史陈列馆3A级和卧牛湖景区2A级质量等级评定。积极协调俄方，加强景区景点建设，提高接待能力。抓好旅游商品开发，带动相关产业。打造旅游精品线路。重点推进“中俄边境第一游”、“世界著名火山游”、“瑷珲历史古城游”、“鄂族民俗风情游”、“俄罗斯腹地观光游”等特色旅游产品开发。加大整体包装力度，开发设计多条国际与国内相结合的特色旅游线路，实现边境游与瑷珲游、火山游及其他国内游有效对接。开拓客源市场。选择国内外重点客源地区，通过媒体及旅游节会等形式，建立多元化、多层次、全方位旅游营销网络，提升黑河旅游整体形象。强化本地旅行社与国内外旅行社联合，不断拓展客源市场。加强旅游市场管理。放宽准入政策，吸引外埠企业参与经营。强化行业管理，杜绝恶性竞争。加快导游管理网络化建设，搞好培训，实现从业人员持证上岗。

（五）加大扶持力度，壮大民营经济。突出民营经济战略地位，扩总量，上规模，提质量，增效益。完善发展机制和服务机制。降低市场准入门槛，消除各种隐性障碍，为民营企业创造平等竞争环境。完善信贷担保体系，加大金融扶持力度。重点扶持20户起步较早、基础较好的骨干企业，注重培育起点高、规模大、带动性强、效益好的企业。建立服务体系，抓好企业诚信认证和质量管理，依法保护从业人员私有财产。引导和监督企业遵守国家法律法规，促进企业健康发展。落实扶持政策。坚持“放手发展，积极引导，迅速提高”方针，凡国家法律未明令禁止的领域和行业，一律放开。特别要引导民营经济参与国企改革和基础设施、公用事业建设。支持机关事业单位工作人员、复转军人、大中专毕业生、企业下岗人员和农民发展民营经济。广泛吸引外来投资，努力激发民间资本的投资热情，在全社会形成政府鼓励创业、群众自主创业的良好氛围。提高企业素质。鼓励民营企业向加工型、外向型、科技型、多元服务型方向发展。做大做强一批具有较强牵动作用的民营企业群体，争取更多企业纳入省民营经济发展项目库。引导有条件的民营企业开展资本联合，进行股份制改造，建立现代企业制度，由家族式经营逐步向现代经营模式转变。

（六）加强城乡建设，加速城市化进程。按照边境口岸城市定位和市场经济要求，推进城市建设。树立规划第一思想。依法强化城市规划机构和职能，科学制定并严格执行规划，抓好城市风貌特色定位及相关规划的编制工作。坚持规划和土地集体审批、集中审定原则，增强规划执行的严肃性和科学性。强化规划监察，杜绝违背城市规划和私接乱建行为。抓好城市资产经营。加快土地资产市场化运营，全面推行经营性土地招标拍卖挂牌出让。按照“谁投资、谁经营、谁受益”原则，深化城市建设投融资体制改革，通过产权转让、入股、使用权出让等方式，进行资产运营。加快基础设施和住房建设。多方筹集资金，改善城市基础设施。黑河垃圾处理厂、应急供水工程年内开工建设。继续修建和改造城区道路和人行道，加强城市绿化工作，增加城市公共绿地面积。加速新区开发，兼顾老城区改造，鼓励发展集资建房和合作建房，培育住房消费热点，改善人居环境。提升城市管理水平。对城市管理行政处罚权实行相对集中，建立高效、顺畅的城管体制。推行“门前包保”责任制，对城乡结合部、城市出口等地段进行专项治理。加快推进环卫行业改革，实行市场化运作。从严整顿建筑市场秩序，规范房地产市场开发经营行为，实施有效监督管理。抓好土地市场治理整顿，依法合理使用土地。增加城市人口，扩大市区规模。坚持产业化发展、多渠道投入，加强社区建设和物业管理。开通市区市政服务热线。推进生态市建设。树立“循环经济”理念，继续抓好“无烟城市”创建工作。从严控制污染物排放总量，推广使用清洁燃料。市区并入集中供热面积20万平方米。加强小城镇建设。以现有国家级、省级试点城镇和生态小城镇为重点，逐步完善道路、给排水、供电等设施建设，完善小城镇功能。发展农副产品加工业，适当向小城镇集中，增强小城镇经济实力。

（七）加强环境建设，开创招商引资工作新局面。抓住全省改善环境年、招商引资年的有利时机，改善环境，大力招商，争取引进到位资金同比增长10%以上。营造安商富商环境。把环境建设作为发展经济的根本性、基础性措施，紧紧抓在手上。切实纠正行业不正之风，集中力量整治影响招商环境、阻碍经济发展的重点问题，依法查处曝光典型案件。创新服务方式，完善“六项制度”，实行政府部门服务责任制。建立外商投诉中心和“一站式”便民服务大厅，公开服务项目，简化和规范办事程序，提高服务质量。广泛开展招商活动。落实奖励机制，充分调动各方面招商积极性。面向国内外大财团、大公司、大民营企业，面向省内先进地区和“两江”、“两东”、“一海”等发达地区，面向计划、专利等职能部门和大专院校、重点科研单位，采取会展招商、组团招商、代理招商、以商招商、网上招商等方式，积极引进战略投资者。加强项目库建设。结合东北老工业基地振兴战略实施，依据市场需求，围绕存量资产盘活、矿产和旅游资源开发、城市基础设施建设、农业产业化、境外投资等方向，做实做细项目前期工作。集中人力财力，论证和储备一批质量高、潜力大的项目，扩大项目库规模。

（八）加强财税金融工作，确保财政收支平衡。围绕扩张经济总量，加强财源建设，财政一般预算收入增长6%。狠抓收入管理。加快纳税监控网络建设，扩大电子申报纳税范围，运用信息化手段提高税收管理水平。依法加强税收征管，堵塞跑冒滴漏，做到应收尽收。加大监督和稽查力度，严厉打击偷逃骗抗等涉税违法行为，坚决制止越权减免征收，维护税收秩序，确保完成收入任务。保证重点支出。合理安排支出结构和次序，确保职工工资和三条保障线资金按时足额发放。加大对农业、教育、公共卫生等方面投入。支持国企改革和事业单位改革，保证必要改革成本。按照一般性支出零增长的要求，严格控制人员编制，严禁超标准配车，坚决压缩会议、招待、考察等方面费用。抓好财源项目建设。向省内十强县学习，把财源建设摆到重中之重的位置，同时抓好弱县帮扶工作。完善工作机制，加大前期投入，切实搞好项目筛选、储备、立项和建设。拓宽融资渠道，通过积极向上争取、吸引民间资本、与大企业挂靠联合等方式筹集建设资金。新上一批对市域经济发展具有较大牵动力的项目，促进在建项目尽快达产达效，努力壮大财源基础。深化财税改革。深入研究税制改革、农村税费改革政策，结合当地实际落实好各项措施。继续深化预算管理体制改革，完善部门预算、国库集中收付、政府采购改革。推行以单位开单、银行收款、财政统管为模式的非税收入管理改革。强化金融支持。充分利用有关政策，减轻企业债务负担，增加贷款规模。支持金融机构核呆和清收工作，依法打击恶意逃废债务以及其他金融违法犯罪行为。打造诚信企业，提高金融信用水平。

（九）坚持以人为本，统筹经济社会协调发展。努力建设社会主义精神文明，贯彻依法治市方针，加强民主法制建设，推进三个文明同步发展。实施人才战略，积极吸引、合理使用各类人才。抓好国民经济和社会信息化建设，以信息化带动工业化。加强科技创新体系建设，大力发展高新技术产业和民营科技企业。搭建中俄科技合作平台，推动俄高新技术成果与国内产业发育的结合。积极发展基础教育，鼓励发展民办教育，扩大普通高中招生规模。力争完成齐大黑河分校专升本工作。调整职业教育专业设置和办学方向，培养实用型人才。启动“村村大学生计划”。加快农村中小学危房改造，计划新建校舍3.8万平方米。建立贫困学生助学机制，遏制中小学生辍学。积极抓好非典和禽流感预防控制工作。加强疾病预防控制体系和医疗救治体系建设，市第一人民医院住院部大楼和全市6所疾控中心业务楼要交付使用，建设市级紧急救援中心，启动6所综合医院传染病区和黑河市中医医疗康复中心建设。加强农村卫生工作，改善乡镇卫生院基础设施，开展农村初级卫生保健和合作医疗试点，逐步建立新型农村合作医疗制度。重视文化基础设施建设，抓好黑河市人民艺术剧院、群众艺术馆改扩建工程，筹建黑河中心图书馆。发育文化产业，满足群众文化生活需要。加快广播电视网改造，扩展广电新业务，解决部分偏远农村看电视听广播难问题。举办第三届全市田径运动会。加强“双拥”工作和国防动员工作，争创国家级“双拥”模范城。做好人口和计划生育工作，发展档案、地方志和文博图事业。加强民族宗教工作，大力发展少数民族经济和文化教育事业。

（十）认真解决热点难点问题，维护社会安全稳定。妥善处理改革、发展、稳定关系，努力营造人民群众安居乐业的社会环境。抓好就业和再就业工作。加大新兴产业开发力度，积极发展服务业特别是社区服务业，开发新的就业岗位。落实各项再就业政策，强化服务措施，安置下岗失业人员。大力组织劳务输出，鼓励到省外、境外就业。加大培训力度，为就业、再就业创造条件。完善社会保障体系。继续巩固两个确保，加大基金收缴力度，扩大社会保险覆盖面，实现养老保险、失业保险、城市低保应保尽保和资金社会化发放。抓好做实基本养老保险个人账户、国有企业下岗职工基本生活保险向失业保险并轨、分离企业办社会测算等基础工作。加强政策对接，努力解决城市社会保障难点问题。深化医疗保险改革，全面推行工伤保险制度。完善社会救助体系，出台地方保障办法及相关配套政策。帮扶弱势群体。深入开展城镇“一帮一”活动和农村贫困户包扶工作，关注残疾人就业和生活，广泛寻求增收渠道，努力为弱势群体排忧解难。加强信访工作。坚持“分级负责、归口办理”和“谁主管、谁负责”原则，落实信访和维护稳定领导责任制。建立健全并认真执行案件办结通报和信访包案责任追究等制度。加强社会治安综合治理和安全工作。加强普法教育，积极开展人民调节和法律援助。坚持预防为主，加强综合整治，探索长效机制、快速反应和应急处理机制，防控结合，惩教结合，降低刑事和治安案件发案率。严厉打击境内外敌对势力和“法轮功”邪教组织破坏活动。坚持不懈抓好生产安全、消防安全、交通安全和森林防火工作，避免各类重特大安全事故和森林火灾发生。加强边境管理，减少涉外事件发生，确保边境安宁。

三、加强政府自身建设，全面提高施政水平

认真实践“三个代表”重要思想，努力建设高素质公务员队伍，认真履行职责，不负重托，不辱使命，创造良好业绩。解放思想，与时俱进。按照“创新思想、创新思路、创新机制、创新方法”的要求，不断破除和摒弃旧观念，注重在实践中总结新经验，探索新思路，寻求新突破。紧紧抓住发展这个第一要务，强化战略机遇意识，增强责任感、紧迫感和使命感，以坚韧不拔、奋发有为的精神状态，努力开创各项工作新局面。深化行政管理体制改革。切实把政府职能转到主要为市场主体服务和创造良好发展环境上来，形成行为规范、运转协调、公正透明、廉洁高效的行政管理体制。改革行政审批制度，除少数重大项目和限制类项目外，对于企业不使用政府资金投资建设的项目，一律由审批制改为备案制。全面实行政务公开，凡不涉及国家安全和保密以及法律法规有特殊规定的事项、政府机关做出的具有普遍约束力的行政规定、事关全局的重大决策，都要通过适当形式向社会公开。改进行政管理方式，推行电子政务，提高行政效率。推进政府机构和事业单位改革。科学决策，依法行政。加强对全局性、战略性、前瞻性重大问题的研究，不断提高决策能力。完善重大决策的规则和程序，完善人民建议征集制度，完善与群众利益密切相关的重大事项社会公示制度、社会听证制度、专家咨询制度，提高科学决策水平和公众参与度。坚持依法行政，理顺行政执法体制，加大政府层级监督力度，强化行政复议、行政诉讼和对行政执法的监督。认真贯彻行政许可法，建立健全具体制度和程序；对现行行政许可项目进行全面清理，对实施行政许可的主体资格依法确认和规范；加强对政策性、规范性文件备案审查力度，从源头上监督和纠正违法行为。自觉接受监督。在市委领导下，主动接受人大法律监督和政协民主监督。认真执行人大及其常委会决议决定，主动向人大及其常委会报告工作，高度重视政协的参政议政作用。认真办理人大代表议案、批评、建议和政协委员提案，支持人大代表、政协委员的评议、视察活动。自觉接受群众团体和舆论监督，虚心听取各民主党派、人民团体和社会各界人士意见，拓宽人民群众参政议政渠道。大兴求真务实之风。坚持一切从实际出发，讲实话、办实事、求实绩。不搞虚报浮夸，不做表面文章，不搞政绩工程、形象工程。精简会议和文件，深入实际，调查研究，主动为基层、为企业、为群众服务。继续落实“两风”建设措施，坚持把抓落实作为转变作风的关键环节，各项工作要细化目标，分解任务，落实责任，务求实效。切实维护群众利益。按照国务院廉政会议要求，认真清理，集中整治，坚决纠正土地征用中侵害农民利益的问题、城镇拆迁中侵害居民利益的问题、企业改革中侵害职工合法权益的问题、拖欠和克扣农民工工资的问题。加大力度，治理教育乱收费，纠正医药界不正之风，减轻农民负担，强化安全生产管理和责任追究，切实维护社会各界、各阶层群众的利益。加强政府廉政建设。牢记“两个务必”，坚持勤俭办一切事业。以“制权、管钱、用人”为主线，加强对权力运行的约束，努力从源头上预防和治理腐败。认真落实党风廉政建设责任制，加强对政府工作人员的严格要求、严格教育、严格管理、严格监督。深入持久开展反腐败斗争，加大查处力度，坚决惩治腐败分子。

各位代表，新的一年黑河面临着加快发展的重要机遇。我们要以邓小平理论为指导，全面贯彻“三个代表”重要思想，与时俱进，开拓创新，励精图治，扎实工作，为开创黑河经济社会发展的新局面而努力奋斗！
